# Nanoliposomes as Vehicles for Astaxanthin: Characterization, In Vitro Release Evaluation and Structure

**DOI:** 10.3390/molecules23112822

**Published:** 2018-10-30

**Authors:** Li Pan, Hongyan Wang, Keren Gu

**Affiliations:** Province Key Laboratory of Cereal Resource Transformation and Utilization, Henan University of Technology, Zhengzhou 450001, China; panli215@haut.edu.cn (L.P.); why@haut.edu.cn (H.W.)

**Keywords:** nanoliposomes, astaxanthin, characterization, in vitro release, structure

## Abstract

Astaxanthin was encapsulated in nanoliposomes by a film dispersion-ultrasonic technique using soybean phosphatidyl choline. The astaxanthin-loaded nanoliposomes displayed advantages in the aspects of high encapsulation efficiency and less particle size with a remarkably homodisperse size distribution. Based on X-ray diffraction and differential scanning calorimetry the analysis, it has been demonstrated that there could be interactions of astaxanthin with the lipid bilayer, resulting in the forming of astaxanthin-loaded nanoliposomes. The thermal gravimetric analysis revealed that the thermal stability of astaxanthin after encapsulation in nanoliposomes was remarkably enhanced as compared to astaxanthin alone. Furthermore, encapsulation could greatly enhance the water dispersibility of astaxanthin. This study also confirmed that encapsulation of astaxanthin in nanoliposomes could be an effective way to supply astaxanthin continuously in the body. The effects of astaxanthin incorporation on structural changes of the liposomal membrane were investigated through steady-state fluorescence measurements. This study revealed that the incorporation of astaxanthin into the lipid bilayer decreased membrane fluidity, but increased micropolarity in the membrane within a certain range of astaxanthin concentrations. Additionally, it indicated that the encapsulation of astaxanthin in the lipid bilayer could be applied to modulate the structural properties of membranes.

## 1. Introduction

Astaxanthin, as one of the natural carotenoids, is present in *Haematococcus pluvialis*, yeast, and in hydrobiontes, such as crab, shrimp, and salmon [1]. Astaxanthin possesses unique antioxidant capacity, superior to vitamin E, β-carotene, and coenzyme Q_10_ [2]. In recent years, astaxanthin has attracted great attention in the scientific world owing to its potential physiological functions, including promotion of immunoreaction and liver function [3], remission of oxidative-stress, as well as protection against tumour, inflammation, photooxygenation by ultraviolet light, *Helicobacter pylori* infection, and angiocardiopathy [4,5]. Unfortunately, because of the 11 conjugated double bonds in the structure of astaxanthin, the stability of astaxanthin can be reduced by adverse environmental factors, including heat, radiation, oxygen, alkaline or acidic solutions [6]. The instability of astaxanthin seriously restricts its applications in functional foods, pharmaceutical products, and the cosmetic industry [7]. Moreover, the poor water solubility of astaxanthin greatly reduces its bioavailability, which also has a negative effect on its practical applications. To overcome these defects, encapsulation has been introduced to promote the stability, water-solubility and bioavailability of astaxanthin. As far as we know astaxanthin could be entrapped in polymers [8], embedded in protein foods [9], encapsulated in proteins/carbohydrates [10], embedded in water-soluble saccharides [11,12,13,14], and incorporated into a blend of DNA and chitosan [15].

Obviously, encapsulation is an efficient approach to protect the substances embedded in the core from negative external conditions. Compared with the above encapsulation techniques, liposomes display outstanding advantages, including their capability of encapsulating both hydrophilic and hydrophobic components, target potential, slow-release properties, as well as biocompatibility [16,17]. Liposomes are colloid systems formed by the dispersion of amphiphilic lipids in aqueous solvents through self-assembly, which have similar lipid bilayer structures as cytomembranes. Nonetheless, the applications of regular liposomes (1–100 μm) are seriously limited because of their large particle sizes. Nanoliposomes, the particle size of which are less than 100 nm, is a novel technique to transport bioactive compounds. Due to their nano scale, nanoliposomes have remarkable advantages, involving superior penetration and special targeting properties. Based on the benefits of encapsulation in nanoliposomes, in this work astaxanthin-loaded nanoliposomes (astaxanthin-LN) were prepared by the film dispersion-ultrasonic technique using soybean phosphatidyl choline (SPC).

Recently, although a few studies have investigated the encapsulation of astaxanthin, the molecular interactions of astaxanthin with the lipid bilayer are not clearly understood. To date, there were no detailed reports on the structure of astaxanthin-LN, which is especially related to the influence of astaxanthin incorporation on the structural properties of membranes measured using a fluorescence technique. Thus, it is very essential and crucial to confirm the effects of astaxanthin incorporation on structural changes of the liposomal membrane as this may provide available information for the encapsulation of this and other hydrophobic substances.

Based on the above, the main purpose of the present work was to assess the influence of astaxanthin incorporation on the structural properties of liposome membranes. Combining thin-film with ultrasound treatment and utilizing SPC were adopted to encapsulate astaxanthin. The astaxanthin-LN were characterized through dynamic light scattering (DLS), transmission electron microscopy (TEM), X-ray diffraction (XRD), differential scanning calorimetry (DSC), thermal gravimetric analysis (TGA), and water-solubility studies. The encapsulation efficiency, particle size, morphology, crystalline state, phase property, thermal behavior, and dissolution test of astaxanthin-LN were investigated. The release characteristics of astaxanthin encapsulated in nanoliposomes were also determined. Furthermore, steady-state fluorescence measurements were considered to study effects of astaxanthin incorporation on structural changes of the liposomal membrane.

## 2. Results and Discussion

### 2.1. Characterization of Astaxanthin-Loaded Nanoliposomes

#### 2.1.1. Determination of Encapsulation Efficiency and Particle Size

The encapsulation efficiency is a key indicator to evaluate the application potential of nanoliposomes. The encapsulation efficiency obtained in the present study was 97.49 ± 0.27%, which is higher than that previously reported [18]. The particle size of liposomes directly affects the absorption and release of core material in vivo. Liposomes with less vesicles can promote the accumulation of core materials at the target site and extend their half-life in blood, and can reduce the uptake of liver and spleen. Therefore, it is prominent important to control the particle size of liposomes. In this study, the average particle size of astaxanthin-LN was 80.31 ± 1.80 nm, which is less than that of astaxanthin-LN (85.1–138.8 nm) prepared by Tamjidi et al. [19].

#### 2.1.2. Morphology of Astaxanthin-Loaded Nanoliposomes

**Figure 1:**
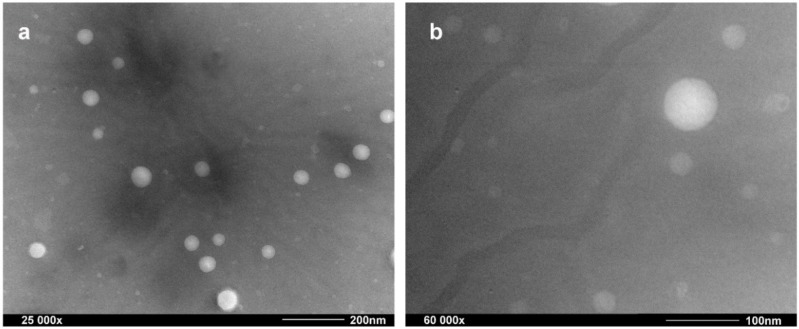
Transmission electron microscope (TEM) micrograph of astaxanthin-loaded nanoliposomes. (**a**) was magnified 25,000×; and (**b**) was magnified 60,000×.

TEM was used to confirm visually the uniformity, size, shape, and intactness of liposome vesicles [20,21]. As seen in Figure 1, it could be observed that astaxanthin-LN vesicles were homogeneous, small, and spherical vesicles without deformations, aggregations or leakage, which are the general characteristics of ideal nanoliposomes. Additionally, the results expressed the particle size of astaxanthin-LN was from 60 to 80 nm with a fairly even size distribution, suggesting that the astaxanthin-LN achieved the desired nanoscale size. This may be attributed to a great mechanical power effect of ultrasound treatment on liposome vesicles [22], which significantly reduces the particle size and improves the uniformity of the size distribution. These results are accordant with that of above particle size measurement using DLS. On the whole, these advantages of astaxanthin-LN could be found in comparison to the previous studies in the aspects of less particle size with a remarkably homodisperse size distribution [23].

#### 2.1.3. X-ray Diffraction Analysis

**Figure 2:**
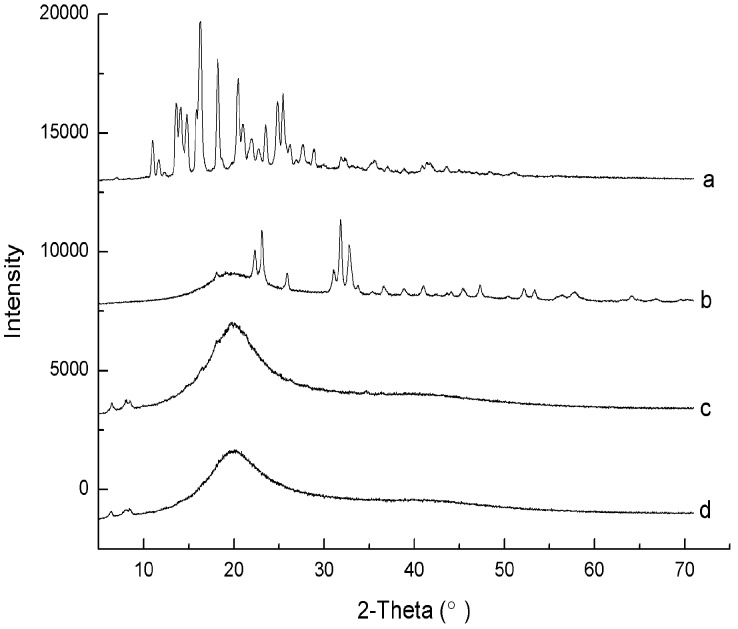
X-ray diffraction (XRD) patterns of astaxanthin (**a**), astaxanthin-loaded nanoliposomes (**b**), soybean phosphatidyl choline (SPC) (**c**) and physical mixture of astaxanthin and SPC (**d**).

The crystalline states of astaxanthin, astaxanthin-LN, SPC, and the physical mixture of SPC and astaxanthin were determined by XRD. There were many crystalline peaks between 5 to 70° (2θ = 16.3, 18.2, 20.5, 22.7, 23.5, 25.5, 31.9, and 32.2°), which could be noted in the diffraction patterns of astaxanthin, as shown in Figure 2a. 

This suggests that astaxanthin mainly exists in a crystalline form. As could be noted in Figure 2c, the patterns of SPC exhibit a large and wide peak, indicating that it has an amorphous character, which agrees well with the results mentioned previously [24,25]. It was obvious that the patterns of the physical mixture was similar to that of SPC, but the characteristic peaks of the astaxanthin disappeared (Figure 2d). Because SPC is a viscous powder, the crystalline form of astaxanthin is overlapped by SPC in the physical mixture. Nonetheless, the characteristic peaks of astaxanthin in the range of 20–35° were seen in the patterns of astaxanthin-LN, confirming the presence of astaxanthin in the nanoliposomes (Figure 2b). Additionally, it could be found that the characteristic peaks of astaxanthin had shifted in the pattern of astaxanthin-LN, which implies that there could be interactions of astaxanthin with the lipid bilayer. It could be also observed that the astaxanthin-LN peaks were different from a simple peak superposition of astaxanthin and SPC, but rather a new phase state. Compared to astaxanthin and SPC, the crystal form of astaxanthin-LN was a mixture of both crystalline and amorphous states, which signified the astaxanthin was successfully encapsulated in the nanoliposomes.

#### 2.1.4. Differential Scanning Calorimetry Analysis

**Figure 3:**
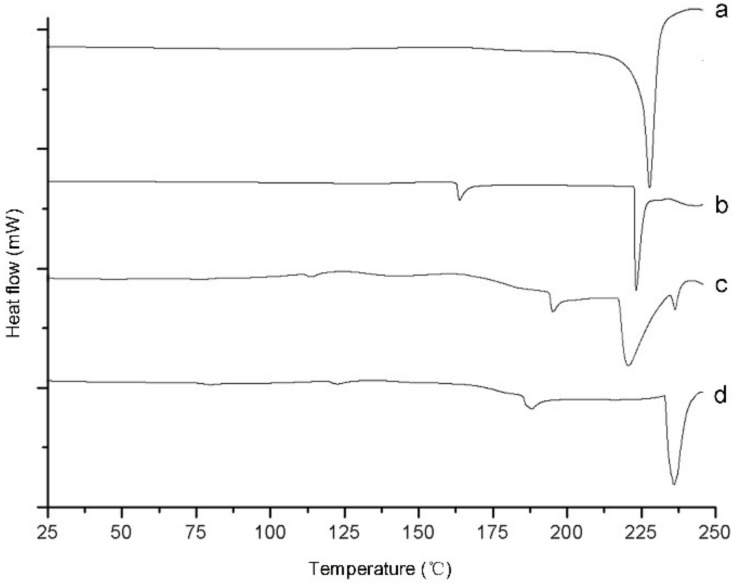
Differential scanning calorimetry (DSC) curves of astaxanthin (**a**); astaxanthin-loaded nanoliposomes (**b**); SPC (**c**) and physical mixture of astaxanthin and SPC (**d**).

DSC was introduced to evaluate the phase characteristics and heat transformations of astaxanthin, astaxanthin-LN, SPC, and the physical mixture of astaxanthin and SPC. As could be seen from Figure 3a, there was an endothermic peak at around 228 °C, which referred to the phase transformation of astaxanthin. The endothermic peaks of SPC were displayed in the thermogram of the physical mixture as shown in Figure 3c,d, in which the endothermic peak of astaxanthin disappeared. Moreover, two peaks were observed in the thermogram of astaxanthin-LN (Figure 3b). The endothermic peak of astaxanthin could also been seen in the DSC curve of astaxanthin-LN, but most of endothermic peaks of SPC had merged with that of astaxanthin. Additionally, the endothermic peak of SPC at around 194 °C shifted to about 163 °C for astaxanthin-LN, which may be ascribed to the hydrogen bonding between astaxanthin and the lipid bilayer. All these results revealed that astaxanthin-LN was formed successfully, which is in agreement with the XRD analysis result.

#### 2.1.5. Thermal Gravimetric Analysis

**Figure 4:**
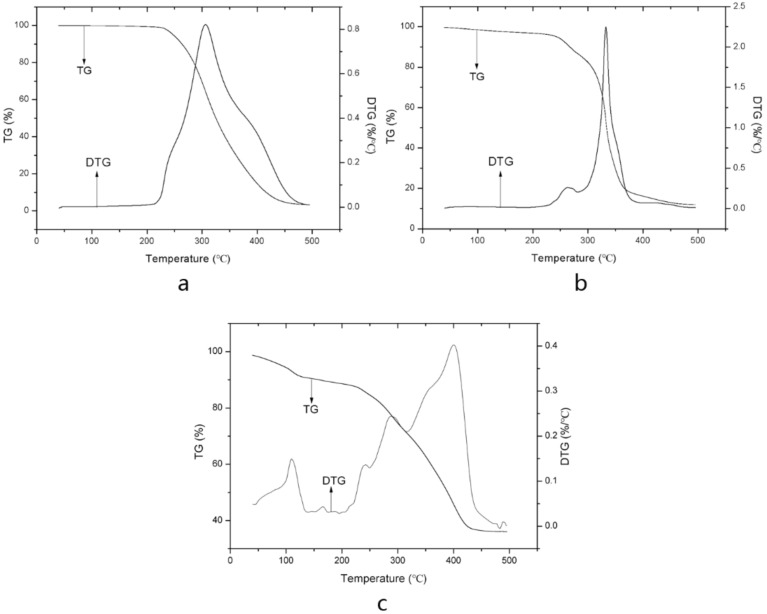
Thermal gravimetric analysis (TGA) curves of astaxanthin (**a**); SPC (**b**); and astaxanthin-loaded nanoliposomes (**c**).

TGA was considered to investigate the thermal behaviors of the samples. The thermogravimetric (TG) and differential thermogravimetric (DTG) curves of astaxanthin, SPC, and astaxanthin-LN are presented in Figure 4. It could be observed that the TG and DTG curves of astaxanthin only have one step or one peak, which reveals that it is a pure compound. A similar result has been reported by Yuan et al. [26]. The decomposition of astaxanthin began at about 220 °C, ending at around 500 °C. The endothermic degradation peak value was around 306 °C. TG and DTG curves of SPC shows two steps. The first step from 200 to 281 °C (with a DTG peak located at 263 °C) was related to the decomposition of SPC. The great weight loss in second step in the temperature range of 281–400 °C was due to the further degradation of SPC, and the DTG peak was at 332 °C. As shown in Figure 4c, the TG and DTG curves of astaxanthin-LN exhibit three steps of decomposition. The first step referred to the evaporation of residual water in astaxanthin-LN. The second step in the range of 138–315 °C shows two main DTG peaks at 241 and 287 °C, which may be attributed to the decomposition of SPC. The incorporation of astaxanthin into the lipid bilayer made SPC degrade at a lower temperature. The third step in the range of 315–500 °C was correlated with the degradation of astaxanthin in the astaxanthin-LN. It could be clearly observed that the starting decomposition temperature of astaxanthin increased from 220 to 315 °C after encapsulation in nanoliposomes, indicating that the encapsulation could effectively enhance the thermal stability of the astaxanthin. Moreover, it could be further demonstrated that astaxanthin was successfully embedded in the nanoliposomes. The result is in accordance with the outcome of studies as reported by Bai et al. [27].

#### 2.1.6. Water Solubility Analysis

The water solubility of astaxanthin-LN and free astaxanthin were investigated. The astaxanthin was adjusted to the same concentration in the two tubes. After the water solubility test, it could be stated that astaxanthin-LN solution had good water dispersibility without insoluble particles or precipitation, but free astaxanthin solution had some insoluble precipitate on the test tube wall. In addition, astaxanthin-LN solution had a darker color than free astaxanthin solution. The solubilities of astaxanthin-LN and free astaxanthin in water were 25.12 μg/mL and 1.45 μg/mL, respectively. Obviously, the solubility of astaxanthin-LN in water was 17 times more than that of astaxanthin. Based on these experimental results, it indicated that encapsulation remarkably improved the water dispersibility of astaxanthin, which may be due to the change in crystal form of astaxanthin after encapsulation in nanoliposomes.

### 2.2. In Vitro Release

**Figure 5:**
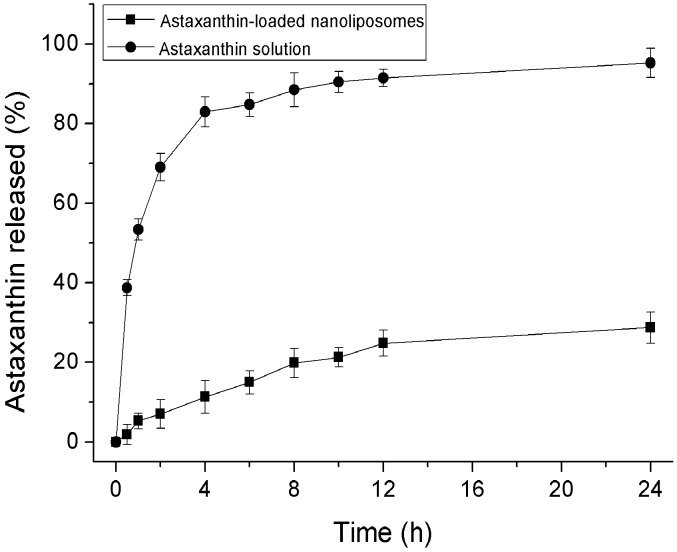
In vitro release of astaxanthin from astaxanthin solution and nanoliposomes in PBS (pH 7.4) at 37 °C. Data are represented as the mean ± standard deviation (*n* = 3).

An in vitro release model can be used to evaluate the release characteristics of nanoliposomes in vitro, which is a crucial index of potential applications of nanoliposomes. The in vitro release characteristics of astaxanthin encapsulated in nanoliposomes and pure astaxanthin solution are depicted in Figure 5. The release of astaxanthin from nanoliposomes was obviously slower in comparison with pure astaxanthin solution. The release rate of astaxanthin from nanoliposomes was 5.32% after 1 h and 19.86% after 8 h, whereas the release rate from pure astaxanthin solution was 53.39% and 88.50%, respectively. After 24 h, the release rate of astaxanthin from nanoliposomes was only 28.74% and from pure astaxanthin solution was 95.27% (Figure 5). It could be noted that nanoliposomes has a controlled release property, which is similar to previous reported studies [28,29]. The controlled release behavior of nanoliposomes may be ascribed to astaxanthin incorporated into lipid bilayer, which inhibits astaxanthin from diffusing too fast into the dialyzate. Thus, the encapsulation of astaxanthin in nanoliposomes could be an effective way to supply astaxanthin continuously in the body.

### 2.3. Effects of Astaxanthin Incorporation on Structural Changes of the Liposomal Membrane

The correct location and orientation of astaxanthin in the membrane, as well as the interactions of astaxanthin and lipid molecule are vital features. The molecular characteristics of astaxanthin in phospholipid membrane were studied using monolayer technique by Shibata et al. [30]. Diverse fluorescence probes have been also introduced to assess the influence of carotenoids on lipid membranes [31].

**Figure 6:**
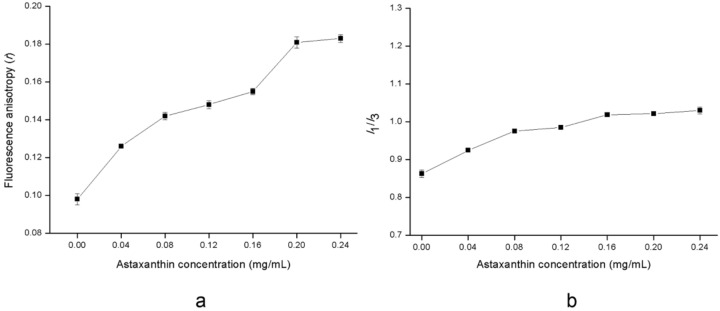
Change in fluorescence anisotropy (*r*) of 1,6-diphenyl-1,3,5-hexatriene (DPH) (**a**) and fluorescence peak intensity ratio of pyrene (*I_1_/I_3_*) (**b**) in membrane as a function of increasing the concentration of astaxanthin. Data are represented as the mean ± standard deviation (*n* = 3).

Fluorescence anisotropy measures the fluorescent depolarization mostly induced by the fluorogen rotator diffusion. As the rotational mobility of liposomes is related to the lipid bilayer, the fluorescence anisotropy could provide vital information about the molecular interactions of astaxanthin with the lipid bilayer. 1,6-diphenyl-1,3,5-hexatriene (DPH) is located in the hydrophobic region of the alkyl chain in the phospholipid membrane. A change in the fluorescence anisotropy of DPH could be noted at 25 °C (Figure 6a) when the concentration of astaxanthin increased. Increasing the concentration of astaxanthin in general resulted in an enhancement in the fluorescence anisotropy, which implies that the fluorescence anisotropy is sensitive to the concentration of astaxanthin. The fluorescence anisotropy of DPH in the lipid bilayer presented an improvement (45.25%) when the concentration increased to 0.20 mg/mL. This may be due to the fact that astaxanthin after incorporation into the lipid bilayer filled the gaps in the alkyl chain of phospholipids and increased the resistance to mobility for DPH in the membrane, which resulted in firmness of the membrane structure. For higher concentrations of astaxanthin, the fluorescence anisotropy stabilized when the concentration increased to 0.24 mg/mL. This indicated that astaxanthin did not affect the order and dynamics in the membrane above a certain concentration. Fluorescence anisotropy measurements revealed that the incorporation of astaxanthin reduced the rotational mobility of the phospholipid axis and made the lipid bilayer to be more ordered (rigid). Thus, these results clearly support a reduction in the membrane fluidity caused by incorporating the astaxanthin.

The ratio of fluorescence intensities at the first (374 nm) and third (391 nm) emission peaks (*I_1_/I_3_*) in pyrene emission spectra could provide a measurement on the polarity of the surroundings. An increase in *I*_1_/*I*_3_ ratio serves as a good indicator of an enhancement of polarity. Figure 6b shows a change in the *I_1_/I_3_* ratio in pyrene emission spectra in membrane with increasing the concentration of astaxanthin at 25 °C. An increase (15.30%) in *I_1_/I_3_* ratio could be noted when concentration of astaxanthin was 0.16 mg/mL, which could signify a modest increase in the micropolarity experienced by the probe with increasing concentration. When the astaxanthin was embedded into the nonpolar region of the lipid bilayer, the nonpolar interspace might be reduced which resulted in less pyrene in the nonpolar area. It was reported that the concentration of pyrene probe in nonpolar or polar regions of a lipid bilayer had an important effect on the polarity [32]. Hence, there was an enhancement in the micropolarity induced by more pyrene in the polar region with increasing the concentration, which agrees with the results as reported by Qiu et al. [33]. However, this effect stabilized with increasing concentration of astaxanthin up to 0.16 mg/mL. This may be ascribed to the saturation of astaxanthin in lipid bilayer beyond its concentration i.e., 0.16 mg/mL.

## 3. Materials and Methods 

### 3.1. Materials

SPC (98%) was bought from Shenyang Tianfeng Biopharming Co., Ltd. (Shenyan, China). Astaxanthin (purity = 98%) was purchased from Shanghai Yanyu Trading Co., Ltd. (Shanghai, China). DPH (purity = 98%) and pyrene (purity = 99%) were obtained from Sigma Chemical Co. (St. Louis, MO, USA). All other solvents used were analytically pure.

### 3.2. Preparation of Astaxanthin-Loaded Nanoliposomes

Astaxanthin-LN were prepared using the film dispersion-ultrasonic technique. An amount of 1 mg of astaxanthin and lipid materials including SPC and cholesterol (5:1, *w*/*w*) were dissolved in 5 mL of chloroform. The emulsion was subjected to rotary evaporation under reduced pressure to remove chloroform, followed by hydratization using 25 mL of 0.05 mol/L phosphate buffer solution (PBS, pH 7.4) under vortexing for 30 min at 50 °C. The suspension was then homogenized in an ice bath for 4 min at 100 W with a probe ultrasonic processor (JY96-IIN, Ningbo Xinzhi Biotechnology Co., Ltd., Ningbo, China). The astaxanthin-LN were moved in a brown-vial brimming with N_2_ and reserved at 4 °C in the dark.

### 3.3. Characterization of Astaxanthin-Loaded Nanoliposomes

The astaxanthin-LN were characterized by DLS, TEM, XRD, DSC, TGA, and dissolution study. The encapsulation efficiency, particle size, morphology, crystalline state, phase behaviour, thermal behavior, and water-solubility test of astaxanthin-LN were investigated. The physical mixture of astaxanthin and SPC was prepared by mixing astaxanthin with SPC directly, in which the amount of astaxanthin is as same as that of astaxanthin-LN.

#### 3.3.1. Encapsulation Efficiency Measurement

The measurement of encapsulation efficiency was carried out through extraction. Based on our previous method: 400 μL of astaxanthin-LN solution was blended with 5 mL of petroleum ether by vortex for 5 min at 30 °C. The blended sample was centrifuged at 3000 rpm for 5 min for the collection of the supernatant. The above process was repeated twice. The gathered supernatant was placed into a round-bottom flask and then attached to a rotatory evaporator to displace the petroleum ether at 50 °C. Then the residual after evaporation was dissolved with chloroform. The content of free astaxanthin was quantified at 492 nm with an ultraviolet-visible spectrometer (TU-1810, Beijing Puhua General Instrument Co., Ltd., Beijing, China), with chloroform as blank. Each test was carried out in triplicates. The encapsulation efficiency (%) were calculated using the following equations:

(1) $$\mathrm{Encapsulation}\mathrm{efficiency}\left(\%\right)=\frac{\mathrm{total}\mathrm{astaxanthin}-\mathrm{free}\mathrm{astaxanthin}}{\mathrm{total}\mathrm{astaxanthin}}\times 100$$

#### 3.3.2. Particle Size Analysis

Particle size of samples were estimated on DLS (Nano-ZS90, Malvern Instruments, Worcestershire, UK) at 25 °C with the 90° of detector angle. The samples were diluted 200 times in distilled water. Each determination was performed in triplicates.

#### 3.3.3. Morphological Study

The morphological study of astaxanthin-LN was carried out with TEM analysis (HT7700, Hitachi, Tokyo, Japan). Liposome samples were diluted 2-fold using PBS (pH 7.4). A drop of the diluted sample was laid on a copper wire mesh with a supporting film and left for 5 min, followed by dyeing with phosphotungstic acid for 5 min. The redundant liquid was then wiped off with a filter-paper. The mesh after dryness under room temperature was analyzed with TEM [34].

#### 3.3.4. X-ray Diffraction

The crystalline states of astaxanthin, astaxanthin-LN, SPC, and the physical mixture of astaxanthin and SPC were assessed by an X-ray diffractometer (Miniflex600, Rigaku Corporation, Tokyo, Japan) with Cu-K α radiation. Measurements were operated at 35 kV and 20 mA. The XRD spectrums of freeze-drying samples were scaned in the 2θ range of 5–70° at a scaning rate of 5°/min.

#### 3.3.5. Differential Scanning Calorimetry

The thermal behaviors of astaxanthin, astaxanthin-LN, SPC, and the physical mixture including astaxanthin and SPC were estimated using a DSC instrument (Q20, TA, New Castle, DE, USA). The freeze-drying samples (approximately 5 mg) were weighed in aluminum DSC pans. An empty aluminum based pan was utilized as reference. The heating range was 25–245 °C at a heating rate of 10 °C/min under dry N_2_ atmosphere (40 mL/min).

#### 3.3.6. Thermal Gravimetric Analysis

TG and DTG analysis of astaxanthin, astaxanthin-LN, SPC, and the physical mixture containing astaxanthin and SPC were performed with a TGA instrument (Q50, TA, New Castle, DE, USA). About 5 mg of freeze-drying samples were placed into open aluminum pans and heated in the temperature range of 40–500 °C at heating rate of 10 °C/min with N_2_ flow set at 60 mL/min.

#### 3.3.7. Water-Solubility Study

The water-solubility study was carried out on the basis of the improved approach as reported by Zou et al. [35]. Astaxanthin or astaxanthin-LN was mixed with distilled water. This astaxanthin-LN sample was adjusted to have the same concentration of astaxanthin as the free astaxanthin sample. The samples were dissolved under ultrasound for 5 min, followed by centrifugation at 5000 rpm for 10 min. Aliquots of 5 mL of the supernatant was added to 5 mL of chloroform. After vortex and centrifugation at 3000 rpm for 10 min at 25 °C, the under layer comprising the dissolved astaxanthin was then gathered, while the top layer was added to other 5 mL of chloroform and the same process was repeated. The under layer was blended with the previous one and measured at 492 nm with an ultraviolet-visible spectrometer (TU-1810, Beijing Puhua General Instrument Co., Ltd., Beijing, China), where chloroform was employed as blank. Each determination was carried out in triplicate. The solubility was expressed as the content of dissolved astaxanthin in per 1 mL of water.

### 3.4. In Vitro Release

In vitro release studies were carried out with astaxanthin-LN and pure astaxanthin solution. An amount of 10 mL of astaxanthin-LN or pure astaxanthin solution was put into a dialysis bag (MWCO 8000–14,000 Da). The bag was doused into 100 mL of 0.05 mol/L PBS (pH 7.4) at 37 ± 0.5 °C, in an aqueous bath under shake at 100 rpm. 1 mL of the releasing media was took out at predetermined intervals (0.5, 1, 2, 4, 6, 8, 10, 12, 24 h), and the same amount of pre-warmed PBS was supplemented. All the release tests were carried out in triplicates. The concentration of astaxanthin in each sample was measured as mentioned above method (Section 3.3.7). The release rate of astaxanthin can be obtained by the following equation:

(2) $$\mathrm{Release}\mathrm{rate}\left(\%\right)=\frac{\mathrm{released}\mathrm{astaxanthin}}{\mathrm{total}\mathrm{astaxanthin}}\times 100$$

### 3.5. Steady-State Fluorescence Determination

The DPH was dissolved in tetrahydrofuran with its concentration adjusted to a final value of 2 × 10^−3^ mol/L. The DPH solution was then stored at 4 °C in the dark. An amount of 1 mL of astaxanthin-LN was diluted with PBS (pH 7.4) to 10 times, and was then mixed with 0.2 mL of DPH solution. The mixture was kept in the dark at 25 °C for 30 min. The fluorescence anisotropy measurements were carried out with a fluorescence spectrometer (FLS980, Edinburgh Instruments, Edinburgh, UK) with equipped with excitation and emission polarizers. The slits of excitation and emission were fixed to 3 nm. Astaxanthin-LN with DPH was excited at 359 nm and the emission was measured at 426 nm, the emission spectra were scanned from 370 to 500 nm. The steady-state fluorescence anisotropy (*r*) was obtained as follows:

(3) $$r=\frac{I_{VV}\;-\;I_{VH}}{I_{VV}\;+\;2GI_{VH}}$$

where *I_VV_* and *I_VH_* are the fluorescence intensities observed in perpendicular and parallel direction to the excitation light beam [36].

For the measurement of pyrene fluorescence, the pyrene in acetone at a final concentration of 2 × 10^−5^ mol/L was added to to 1 mL of astaxanthin-LN which was diluted with PBS (pH 7.4) to 10 times. The sample was incubated in the dark at 25 °C for 30 min. The excitation wavelength was selected to 339 nm, and the fluorescence intensities were measured at the first (*I*_1_) and at the third (*I*_3_) emission peaks with the fluorescence spectrometer as described above. The slits of excitation and emission were 5 nm. The selected concentrations of astaxanthin (*w*/*v*, mg/mL) were 0.04, 0.08, 0.12, 0.16, 0.20, and 0.24 mg/mL, respectively.

### 3.6. Statistical Analysis

All experiments were performed in triplicates. The results were presented as means ± standard deviation (SD). One-way analysis of variance (ANOVA) test was employed to assess the significant statistical difference (*p* < 0.05) with SPSS software (version 23.0, IBM Corporation, New York, NY, USA).

## 4. Conclusions

In the current investigation, astaxanthin-LN could be prepared by the film dispersion-ultrasonic technique using SPC. The astaxanthin-LN displayed adventages in the aspects of high encapsulation efficiency and less particle size with a remarkably homodisperse size distribution compared with previous studies. XRD and DSC analyses demonstrated that there could be interactions between astaxanthin and the lipid bilayer, which resulted in the formation of astaxanthin-LN. According to the TGA results, the thermal stability of the astaxanthin could be effectively improved after encapsulation in nanoliposomes. In addition, water-solubility analysis revealed encapsulation could remarkably enhance the water dispersibility of astaxanthin. This study also suggested that release rate of astaxanthin could be reduced by encapsulating with nanoliposomes. The effects of astaxanthin incorporation on structural changes of the liposomal membrane was evaluated through steady-state fluorescence measurements. Astaxanthin incorporation into the lipid bilayer decreased the membrane fluidity, but increased the micropolarity in membranes. Moreover, these results showed that the membrane structure was stabilized when the lipid bilayer was saturated with astaxanthin. The encapsulation of astaxanthin in the lipid bilayer could be adapted to regulate the structural properties of membranes. These results may guide the development of nanoliposomes as an effective carrier of hydrophobic substances in molecules, pharmacy, and food.
